# Catalytic Effects in Lithium–Sulfur Batteries: Promoted Sulfur Transformation and Reduced Shuttle Effect

**DOI:** 10.1002/advs.201700270

**Published:** 2017-09-05

**Authors:** Donghai Liu, Chen Zhang, Guangmin Zhou, Wei Lv, Guowei Ling, Linjie Zhi, Quan‐Hong Yang

**Affiliations:** ^1^ School of Chemical Engineering and Technology and Collaborative Innovation Center of Chemical Science and Engineering (Tianjin) Tianjin University Tianjin 300072 China; ^2^ School of Marine Science and Technology Tianjin University Tianjin 300072 China; ^3^ Department of Materials Science and Engineering Stanford University Stanford CA 94305 USA; ^4^ Engineering Laboratory for Functionalized Carbon Materials and Baotou Graphene Innovation Center (Shenzhen) Graduate School at Shenzhen Tsinghua University Shenzhen 518055 China; ^5^ National Center for Nanoscience and Technology Beijing 100190 China

**Keywords:** catalytic effect, confinement, Li–S batteries, shuttle effect

## Abstract

Lithium–sulfur (Li–S) battery has emerged as one of the most promising next‐generation energy‐storage systems. However, the shuttle effect greatly reduces the battery cycle life and sulfur utilization, which is great deterrent to its practical use. This paper reviews the tremendous efforts that are made to find a remedy for this problem, mostly through physical or chemical confinement of the lithium polysulfides (LiPSs). Intrinsically, this “confinement” has a relatively limited effect on improving the battery performance because in most cases, the LiPSs are “passively” blocked and cannot be reused. Thus, this strategy becomes less effective with a high sulfur loading and ultralong cycling. A more “positive” method that not only traps but also increases the subsequent conversion of LiPSs back to lithium sulfides is urgently needed to fundamentally solve the shuttle effect. Here, recent advances on catalytic effects in increasing the rate of conversion of soluble long‐chain LiPSs to insoluble short‐chain Li_2_S_2_/Li_2_S, and vice versa, are reviewed, and the roles of noble metals, metal oxides, metal sulfides, metal nitrides, and some metal‐free materials in this process are highlighted. Challenges and potential solutions for the design of catalytic cathodes and interlayers in Li–S battery are discussed in detail.

## 1 Introduction

Among the state‐of‐the‐art energy storage devices, the lithium–sulfur (Li–S) battery is a promising candidate for next‐generation batteries because of its high theoretical energy density (≈2600 Wh kg^−1^), and the low cost and environmental friendliness of the sulfur cathode material.^1^ Despite these advantages, many challenges have to be overcome in its commercialization, such as the poor utilization and huge volume change (80%) of sulfur and its slow reaction kinetics. Highly soluble intermediate lithium polysulfides (LiPSs) are formed during the cycling which are dissolved into the electrolyte and some LiPSs shuttle from the cathode to the anode to form non‐reusable solid Li_2_S_2_/Li_2_S.^2^, ^3^ This leads to a continuous loss of active material, passivation of the anode, and low coulombic efficiency, and thus, it is considered to be one of the key issues limiting the practical use of Li–S batteries.^4^ Many efforts have been made to solve the shuttle effect that is to suppress the diffusion of LiPSs.^3^, ^5^, ^6^ Various carbon materials have been used as the sulfur host and conductive framework in Li–S batteries because of their high conductivity and large surface area.^3^, ^6^, ^7^ However, carbon materials always have a nonpolar surface, which leads to their relatively low affinity for polar LiPSs and does not help restrict LiPS shuttling. Nanostructured inorganic compounds including transition‐metal oxides, sulfides, and carbides have a strong chemical affinity with LiPSs and block the diffusion of LiPSs more efficiently.^8^, ^9^

However, the shuttle effect has not really been well investigated because physical or chemical adsorption only addresses the superficial problem and not the root. The theoretical capacity of an Li–S battery is mostly due to the transformation of soluble long‐chain LiPSs to insoluble low‐chain Li_2_S_2_/Li_2_S.^10^ The soluble LiPSs formed are easily dissolved in the electrolyte, thus losing electric contact with the electrode surface. This greatly decreases the reaction kinetics of the transformation of LiPSs to insoluble Li_2_S_2_/Li_2_S, and even worse, leads to their nonuniform deposition on the electrode surface, forming large Li_2_S_2_/Li_2_S particles and losing electric contact with electrode. In addition to their high activation energy, these solid products are hard to be reused during the reactions, resulting in an increase of internal resistance and loss of active material. The accumulation of LiPSs in electrolyte also causes severe shuttling of them to the anode to form non‐reusable solid Li_2_S_2_/Li_2_S. Both of them lead to the irreversible loss of active materials forming the “dead sulfur.” Above discussion clearly shows that the shuttle effect is mainly derived from the following two reasons. One is the LiPS dissolution in electrolyte and the other is the slow transformation of these LiPSs. In most cases, to achieve the high activity of sulfur for reaction, the contact between sulfur and electrolyte is needed and the LiPS dissolution is hard to be avoided. Thus, the most promising way to restrain the shuttling is to promote the LiPS conversion to solid Li_2_S_2_/Li_2_S to decrease their dissolution in electrolyte. Recently, studies have shown that some polar hosts, such as metal oxides,^11^ metal sulfides,^12^ metal nitrides,^13^ and some metal‐free materials, not only possess strong affinity toward LiPSs but also catalytically promote the conversion of LiPSs to Li_2_S_2_/Li_2_S, indicating a promising way to finally suppress the LiPS shuttling.

**Figure 1:**
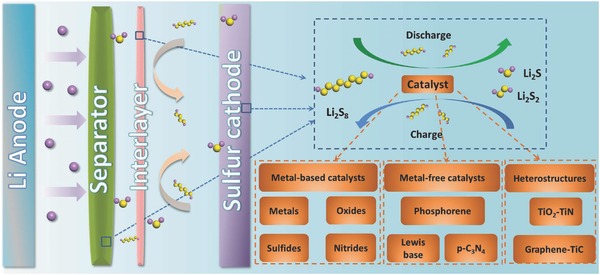
Illustration of catalytic effects in Li–S batteries for the fast conversion of polysulfides to insoluble Li_2_S_2_/Li_2_S, and vice versa.

In the reverse reaction, the transformation of insoluble Li_2_S_2_/Li_2_S back to LiPSs needs a large activation energy, which is made worse by their aggregation during their formation process, leading to slow reaction kinetics and low energy efficiency. Moreover, the formed insoluble Li_2_S_2_/Li_2_S has a high electronic resistivity that increases the internal resistance.^14^ Therefore, increasing the rate of transformation of the captured LiPSs to insoluble Li_2_S_2_/Li_2_S, and vice versa, is key to suppress the LiPS shuttling and developing a practically useable Li–S battery (**Figure** **1**). Until now, several studies have shown that the catalytic effect of some polar hosts in Li–S system accelerates the above redox reactions, which is promising in solving the shuttling problem. Here, recent advances on ways to increase the reaction kinetics of an Li–S battery using a designed catalytic process are reviewed, and comments are made on future challenges and prospects for high‐performance material design.

## 2 Catalytic Metal‐Based Hosts for Li–S Batteries

In the context of electrochemistry, various metals and metal‐containing compounds are often used to increase the electrochemical reaction rates because of their high catalytic activity. For example, a common electrocatalyst is platinum nanoparticles (NPs) supported on carbon particles.^15^ The platinum increases the rate of oxygen reduction either to water, or to hydroxide or hydrogen peroxide in a fuel cell. In the Li–S battery, these materials can also be used as LiPSs hosts due to their abundant polar active sites,^16^ which produce chemical binding of the LiPSs.^9^ Recently, they have been found to have a catalytic effect to achieve fast and efficient conversion of LiPSs to Li_2_S_2_/Li_2_S, and vice versa, and this is reviewed in this section.

### 2.1 Metals

**Figure 2:**
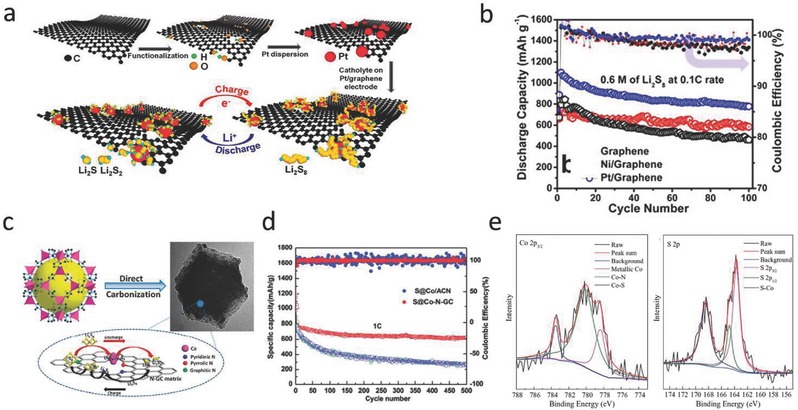
a) Illustration of a Pt electrocatalyst anchored on graphene and its interaction with LiPSs during the charge/discharge process. b) Cycling performance and coulombic efficiency of the hybrids of pristine graphene and graphene with anchored Pt electrocatalyst particles with sulfur. Reproduced with permission.^18^ Copyright 2015, American Chemical Society. c) Illustration of Co‐N‐GC and its interaction with LiPSs. d) Cycling performance and coulombic efficiency comparisons between the S@Co‐N‐GC and the S@Co/ACN electrodes at 1 C. Reproduced with permission.^19^ Copyright 2016, Royal Society of Chemistry. e) Co 2p_3/2_ and S 2p X‐ray photoelectron spectroscopy (XPS) of the as‐prepared S/GN‐CNT composition. Reproduced with permission.^20^

Arava and co‐workers investigated the electrocatalytic effect of the noble metal catalyst, Pt, on the redox reaction of LiPSs.^17^ It has been shown that Pt promotes the redox reaction of LiPSs during the charge/discharge process, but a high surface area host is needed to help adsorb the soluble LiPSs. Thus, they used a graphene host decorated with the Pt catalyst (**Figure** **2**a),^18^ and the prepared Pt/graphene hybrid with sulfur delivered a capacity of 1100 mAh g^−1^ for the initial discharge process that remained at 789 mAh g^−1^ after 100 cycles (superior to the referenced Ni/graphene, 740 mAh g^−1^ for the initial discharge process and 580 mAh g^−1^ after 100 cycles) (Figure 2b). The catalytic effect of Pt on the charge transfer kinetics was revealed by the Tafel results, which showed that the exchange current density of the Pt/graphene was much higher than pristine graphene, suggesting an increase in the rate of LiPS conversion. It was also shown that Pt promoted the conversion of Li_2_S_2_/Li_2_S into long‐chain LiPSs without allowing their aggregation on the electrode.^18^

Besides this noble metal, the transition metals also have a high catalytic activity in many electrochemical reactions. Dong and co‐workers obtained a 3D porous N‐doped graphitic carbon‐Co hybrid (Co‐N‐GC) through the thermal carbonization of a metal–organic framework as shown in Figure 2c.^19^ Co promotes the conversion of Li_2_S_2_/Li_2_S to soluble LiPSs and the transformation of soluble long‐chain LiPSs to insoluble Li_2_S_2_/Li_2_S. Moreover, the N‐containing groups in Co‐N‐GC significantly increase its adsorption energy for Li_2_S*_n_* (*n* = 4–8), which facilitates the oxidization of Li_2_S_6_ to S_8_. With a high sulfur content of 70%, the formed hybrid delivers a high specific capacity of 1670 mAh g^−1^ and maintains a stable cycling performance for 500 cycles under 1 C (Figure 2d). Recently, Gao and co‐workers also reported the catalytic effect of Co on promoting transformations between LiPSs and Li_2_S_2_/Li_2_S.^20^ After introducing sulfur into a 3D graphene–carbon nanotube matrix containing Co NPs, the X‐ray photoelectron spectroscopy (XPS) spectra indicate a strong chemical interaction of the metallic Co NPs with sulfur species (Figure 2e). This hybrid delivers a high capacity of 1049.6 mAh g^−1^ for the initial cycle and 639 mAh g^−1^ after 200 cycles under 0.1 C.

The above results show that metal catalysts greatly enhance the electrochemical redox reaction kinetics. More importantly, these metals have a high electronic conductivity, which leads to a significant improvement in the sulfur utilization. However, these catalysts are either costly noble metals or heavy metals, which are not favorable for practical applications. Besides, the utilization of metal catalysts needs to be further improved through increasing their active surface area according to the catalyst design principles in traditional electrochemical processes. Thus, metal catalysts that cooperate with high surface area carbon materials are more appealing candidates for high‐performance electrocatalysts in Li–S batteries.

### 2.2 Oxides

**Figure 3:**
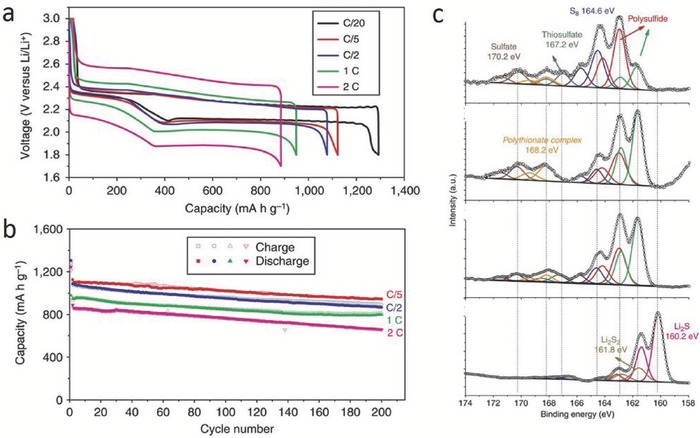
a) Voltage profiles of S/MnO_2_ nanosheets at different current densities ranging from C/20 to 2 C. b) Cycling performance of S/MnO_2_ nanosheets at different current densities. c) Ex situ XPS of S/MnO_2_ nanosheet electrodes after discharge to specific states: from top to bottom: discharge to 2.15 V, discharge to 2.15 V and then aged in the cell for 20 h, discharge to 800 mAh g^−1^ and discharge to 1.8 V. Reproduced with permission.^21^ Copyright 2015, Nature Publishing Group.

Compared with the above metal catalysts, the low‐cost transition metal oxides, such as MnO_2_ and Fe_3_O_4_, also show a catalytic effect on the conversion of LiPSs. Nazar and co‐workers reported the promoted conversion of LiPSs to insoluble Li_2_S_2_/Li_2_S on an ultrathin MnO_2_ nanosheet (NS) surface.^21^ In their work, the MnO_2_ NSs react with LiPSs, forming surface‐bound intermediates, and then these intermediates interact with soluble LiPSs generating polythionates and forming insoluble Li_2_S_2_/Li_2_S. This process is confirmed by XPS (**Figure** **3**c) in which the existence of thiosulfate (167.2 eV) and polythionate (168.2 eV) during the reaction is identified, suggesting that MnO_2_ plays a vital role in the LiPSs transformation. The S/MnO_2_ hybrid (75S/MnO_2_: sulfur content 75 wt%) shown in Figure 3a,b, delivers capacities of ≈1300 mAh g^−1^ at a current density of C/20, and 1030 mAh g^−1^ after 200 cycles. However, the low conductivity of MnO_2_ is not beneficial in improving the sulfur utilization and rate performance, and thus the incorporation of highly conductive materials is normally needed. Cai and co‐workers reported that ultrafine La_2_O_3_ NPs decorated on a nitrogen‐doped mesoporous carbon can not only trap LiPSs, but also show catalytic effect on sulfur reduction, revealing the great potential as the sulfur host to realize high capacity and rate performance.^22^

**Figure 4:**
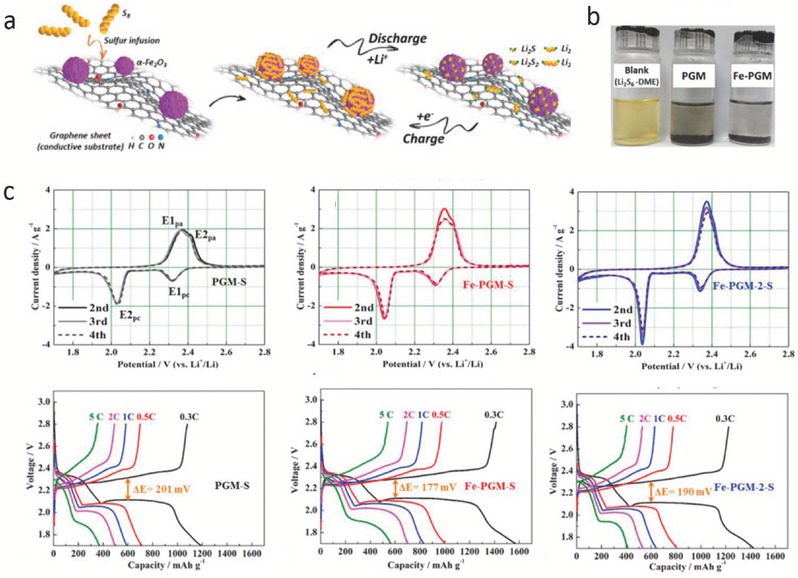
a) Schematic of the conversion process of sulfur on a graphene surface with Fe_2_O_3_ NPs. b) Optical photos of PGM and Fe‐PGM materials soaked in an Li_2_S_6_‐DME solution. c) Electrochemical performance of the Fe‐PGM‐S hybrid compared to those of the PGM‐S and Fe‐PGM‐2‐S hybrids: CV profiles of PGM‐S, Fe‐PGM‐S, and Fe‐PGM‐2‐S at scan rate of 0.1 mV s^−1^ and the charge/discharge profiles of PGM‐S, Fe‐PGM‐S, and Fe‐PGM‐2‐S at various rates ranging from 0.3 to 5 C. Reproduced with permission.^11^ Copyright 2017, Elsevier.

Our group reported that Fe_2_O_3_ NPs promote the transformation of LiPSs into insoluble products and thus restraining the shuttle effect (**Figure** **4**a).^11^ As shown in Figure 4b, a graphene foam containing Fe_2_O_3_ NPs (Fe‐PGM) shows stronger adsorption than a pure graphene foam (PGM), suggesting that the Fe_2_O_3_ NPs have a strong interaction with LiPSs. The cyclic voltammetry and charge/discharge profiles in Figure 4c indicate that Fe_2_O_3_ NPs promote the fast conversion of soluble LiPSs to insoluble Li_2_S_2_/Li_2_S. With an increase in Fe_2_O_3_ content, the E2_pc_ peak shows a higher intensity and becomes narrower, and the E2_pa_ peak shifts to a lower potential indicating better reaction kinetics. The formed cathode material with sulfur delivers a high capacity of 565 mAh g^−1^ with an ultralow capacity fade of 0.049% per cycle over 1000 cycles at 5 C.

Different from MnO_2_ and Fe_3_O_4_, Ti_4_O_7_ has a high conductivity (larger than 10^3^ S cm^−1^ at room temperature) and excellent thermal stability.^23^ Nazar and co‐workers found the Magnéli phase Ti_4_O_7_ showed a strong chemical affinity for LiPSs and promoted the reduction of LiPSs to Li_2_S.^24^ They used an operando X‐ray absorption near‐edge structure to explore the interaction between Ti_4_O_7_ and LiPSs, and the results showed that the LiPSs chemically adsorbed on the Ti_4_O_7_ host were much more easily converted into Li_2_S than those on the carbon surface. In other words, the Ti_4_O_7_ host produced a faster transformation process from LiPSs to Li_2_S avoiding their dissolution in the electrolyte. As a result, the formed hybrid (sulfur content: 60 wt%) shows good a capacity retention over 250 cycles at 0.5 C with a fade rate as low as 0.08% per cycle.

The above studies show that some metal oxides promote the transformation of soluble LiPSs to insoluble Li_2_S_2_/Li_2_S, but most of them have poor conductivity, which increases the battery resistance and lowers the rate capability. Although Ti_4_O_7_ is more appealing due to its high conductivity, its preparation process is complex and costly. Thus, efforts need to be made to improve the conductivity of low‐cost metal oxides or coupling oxides with conducting networked substrates like carbon materials.

### 2.3 Sulfides

**Figure 5:**
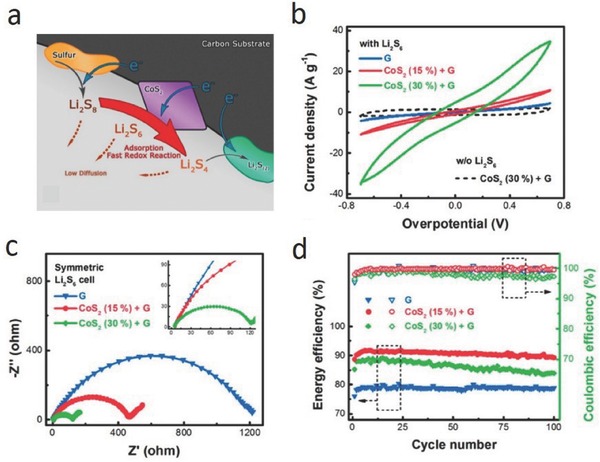
a) Illustration of the discharge process in a CoS_2_‐incorporated carbon/sulfur cathode. Dynamically enhanced polysulfide redox by CoS_2_. b) Polarization curves and c) EIS spectra of symmetrical Li_2_S_6_–Li_2_S_6_ cells and d) energy efficiency and coulombic efficiency of graphene, CoS_2_ (15%) + G, and CoS_2_ (30%) + G‐based sulfur cathodes at a current density of 0.5 C. Reproduced with permission.^28^ Copyright 2015, American Chemical Society.

**Figure 6:**
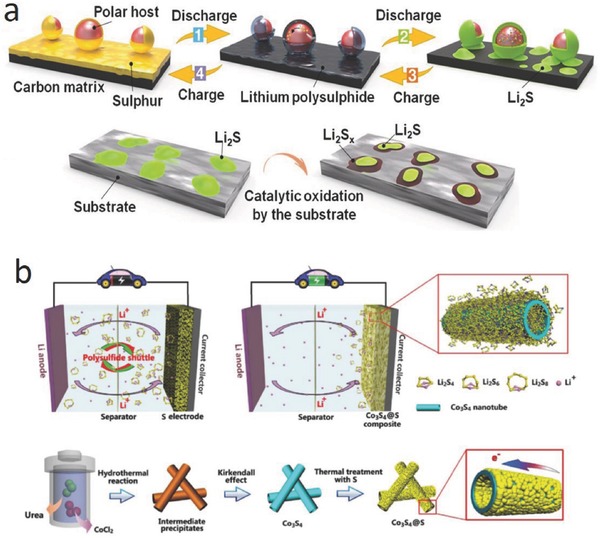
a) Illustration of the sulfur conversion process and the Li_2_S catalytic oxidation on the surface of the substrate. Reproduced with permission.^12^ Copyright 2017, National Academy of Sciences. b) Illustration of a Co_3_S_4_@S nanotube composite to minimize the problems associated with the polysulfides and the fabrication procedure of Co_3_S_4_@S nanotubes. Reproduced with permission.^32^ Copyright 2017, Elsevier.

Transition metal sulfides are used as catalysts for the hydro‐desulfurization of fuels and corrosion protection.^25^ In an Li–S battery, in addition to their strong chemical interaction with LiPSs, recent studies found that metal sulfides had strong catalytic activity in promoting the redox reactions of LiPSs and Li_2_S_2_/Li_2_S. Pyrite cobalt disulfide (CoS_2_) is a typical electrocatalyst in many applications,^26^ and Jin and co‐workers found that CoS_2_ exhibited a high electrocatalytic activity for polysulfide reduction in quantum dot‐sensitized solar cells.^27^ Inspired by those studies, Zhang and co‐workers added CoS_2_ to a carbon/sulfur cathode in an Li–S battery,^28^ so that the interaction between CoS_2_ and LiPSs would accelerate the redox reactions of LiPSs (**Figure** **5**a), greatly improving the performance of the battery. Cyclic voltammograms (CV) tests for the symmetrical Li_2_S_6_–Li_2_S_6_ cells shown in Figure 5b (a CoS_2_ and graphene mixture was used for the working, counter electrode) indicate that the current density increased by an order of magnitude as the CoS_2_ content increased from 0 to 30 wt%. Electrochemical impedance spectra (EIS) of the symmetrical cells (Figure 5c) indicate that charge transfer at a CoS_2_–LiPSs interface is much faster than that at a graphene–LiPSs interface. These electrochemical results suggest an increase of the electrochemical reaction kinetics with the help of CoS_2_. Cui and co‐workers found that metal sulfides accelerate the oxidation of Li_2_S to sulfur (**Figure** **6**).^12^ Electrochemical measurements and XPS studies show that VS_2_, CoS_2_, and TiS_2_ have higher binding energies toward Li_2_S_6_. First‐principles calculations show that the strong interaction between LiPSs and metal sulfides lowers the overpotential for the Li_2_S decomposition. The prepared hybrid materials of S‐VS_2_@G/CNT, S‐CoS_2_@G/CNT, and S‐TiS_2_@G/CNT, respectively, deliver high capacities of 1093, 1033, and 1008 mAh g^−1^ at 0.2 C, and excellent cycling stabilities with low capacity decay rates of 0.070%, 0.084%, and 0.088% even at 2 C. This work provides a fundamental understanding of the catalytic process and also guides the rational design of host materials for a high capacity and long‐life Li–S battery.

Cui and co‐workers found that the edge sites of MoS_2_ show much stronger interaction with Li_2_S than the terrace counterpart, which also have high electrochemical selectivity and activity for Li_2_S deposition.^29^ Arava and co‐workers also found that edge sites of atomically thin WS_2_ and MoS_2_ NSs can lower the polarization and enhance the electrochemical reaction kinetics.^30^ Goodenough and co‐workers recently reported that the dangling sulfur bonds on the edges of WS_2_ show strong adsorption ability toward LiPSs.^31^ Moreover, the sulfophilic WS_2_ enhances the transformation of trapped LiPSs to Li_2_S and thus lowers the internal resistance. Pan and co‐workers recently reported the use of highly conductive Co_3_S_4_ as the host material for high performance Li–S battery.^32^ Co_3_S_4_@S nanotube cathodes show relatively fast electrochemical reaction kinetics, as indicated by EIS and CV results. Lee and co‐workers showed that sulfur deficiencies on the surface of MoS_2_ participate in the LiPS conversion process and improve the kinetics of the LiPSs redox reaction, which decreases the accumulation of LiPSs and thus leads to improved performance.^33^ However, in the above studies, the transition metal sulfides are usually present as large particles, and the design of the nanostructure and the number of catalytic active sites still needs optimization to achieve high sulfur loaded cathodes for high‐energy density Li–S batteries.

### 2.4 Nitrides

**Figure 7:**
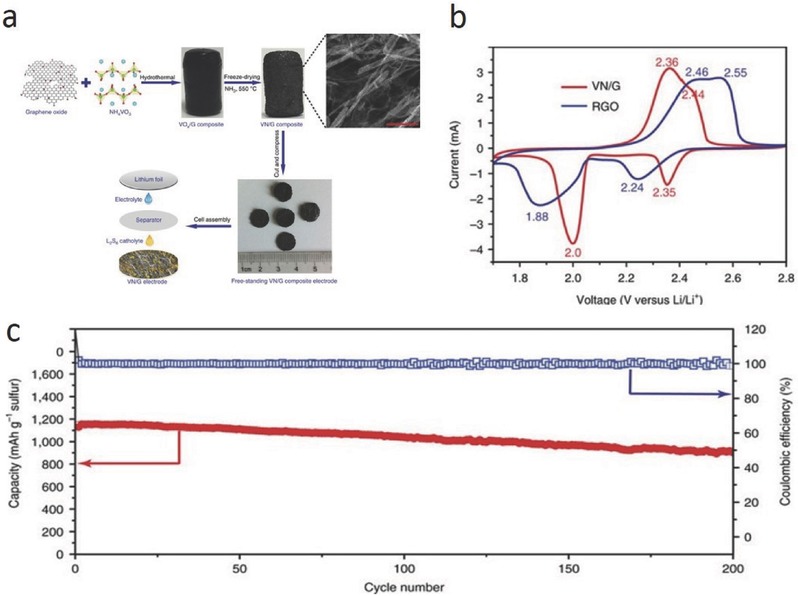
a) Schematic of the fabrication process of a VN/G composite and the cell assembly. b) CV profiles of the VN/G and RGO cathodes at a scan rate of 0.1 mV. c) Cycling performance of the VN/G cathode at 1 C. Reproduced with permission.^13^ Copyright 2017, Nature Publishing Group.

Transition metal nitrides have already been widely investigated in supercapacitors and lithium‐ion batteries because of their high conductivity.^34^ Recently, Mosavati et al. showed that the use of WN as a sulfur host led to an excellent electrochemical performance.^35^ XPS results indicated that WN traps LiPSs due to the formation of S—W—N bonds, which effectively enhance the electrochemical reaction kinetics. Li and co‐workers synthesized a conductive porous VN nanoribbon/graphene (VN/G) hybrid as the sulfur host (**Figures** **7**a).^13^ The polar VN shows a strong chemical affinity for soluble LiPSs and restrains their shuttling. The CV profile shows that the introduction of VN leads to higher reduction peaks and lower oxidation peaks, suggesting improved redox kinetics (Figures 7b). When used as the cathode material of an Li–S battery, the VN/graphene/sulfur cathode delivers the capacity of 1471 mAh g^−1^ for the initial discharge progress at 0.2 C. Even at a high rate of 1 C, it still delivers a high capacity of 1128 mAh g^−1^, which remains at 917 mAh g^−1^ after 200 cycles (Figure 7c). Kim and co‐workers reported that long‐chain LiPSs can be fragmented into short‐chain LiPSs on the surface of TiN, which has been proved with the computational and experimental analyses.^36^ Such a catalytic effect is derived from the ultrastrong chemical bonding between elemental sulfur and the TiN surface.

The biggest advantage of the transition metal nitrides, such as WN, VN, and TiN, is their high conductivity. The strong chemical interaction between metal nitrides and LiPSs helps alleviate the formation of insoluble Li_2_S_2_/Li_2_S on the cathode surface and further reactivates insoluble Li_2_S_2_/Li_2_S to LiPSs. However, the preparation of metal nitrides often needs high‐temperature treatment under an NH_3_ atmosphere, and the active surface area needs to be optimized to improve the catalytic efficiency.

## 3 Metal‐Free Polar Materials with Catalytic Effects in Li–S Batteries

**Figure 8:**
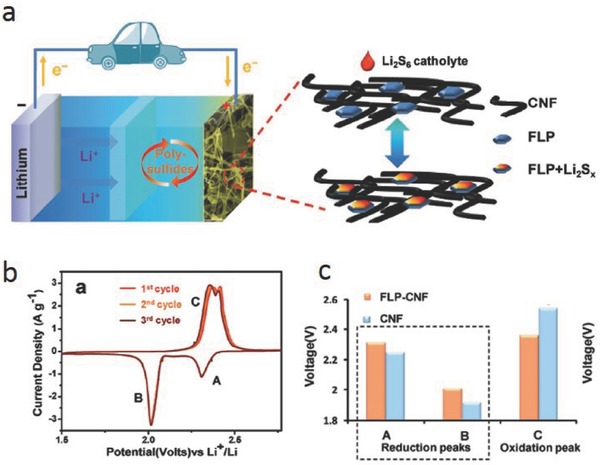
a) Schematic of the FLP‐incorporated carbon nanofiber (CNF) matrix used as the host for the LiPSs catholyte. b,c) CV test and onset potential of FLP‐CNF and pure CNF electrodes to study the kinetics of the electrochemical reactions in an Li–S battery. Reproduced with permission.^38^

Recently, some metal‐free materials that are rarely used as catalysts have been reported to show catalytic effects in promoting the conversion of soluble LiPSs to insoluble Li_2_S_2_/Li_2_S and vice versa. Phosphorene is an atomically thin sheet and its use as the active electrode material in an Li–S battery has been proposed,^37^ and showed excellent electrochemical performance. Recently, Koratkar and co‐workers introduced few‐layer phosphorene (FLP) NSs, which were embedded in the porous carbon nanofiber networks in Li–S batteries (**Figure** **8**a).^38^ Phosphorene significantly lowered the electrode polarization and accelerated the redox reaction which was proved by the CV and the onset potential in Figure 8b,c. Highly reversible reactions of a battery containing FLP were also achieved.

N‐doped carbons have been proposed as nonmetallic catalysts especially for the electrocatalysis process.^39^ Dong and co‐workers introduced N‐doped carbon as a conductive Lewis base matrix into the cathode and investigated its ability to improve the electrochemical performance.^40^ This work showed that the N‐doped carbon surface serves as a conductive Lewis base “catalyst” matrix to increase the adsorption energy for Li_2_S*_n_* (*n* = 4–8) and fill up the gap of Li_2_S_6_ into S_8_ to complete the sulfur–LiPSs redox routes. This significantly improved the sulfur utilization and the cyclic stability. The Δ*E* between the charge and the discharge profiles was also greatly reduced, suggesting greatly decreased polarization and improved reaction kinetics.

Polymeric carbon nitride (p‐C_3_N_4_) has been widely investigated as a photocatalyst due to its chemical stability, tunable electronic structure, and high surface polarization.^41^ Li and co‐workers demonstrated a strong electrostatic affinity between p‐C_3_N_4_ and LiPSs, which not only stabilized the cycling stability, but also increased the redox kinetics during the charge/discharge process.^42^ Density functional theory (DFT) calculations show that LiPSs are adsorbed on the p‐C_3_N_4_ surface by an electrostatic interaction, and this serves as a good immobilizer for LiPSs. p‐C_3_N_4_ was integrated with reduced graphene to prepare a sulfur host, which led to a low polarization as well as a low kinetic barrier in the electrochemical redox of LiPSs.

## 4 Heterostructures to Realize a Smooth Trapping–Diffusion–Conversion of LiPSs

**Figure 9:**
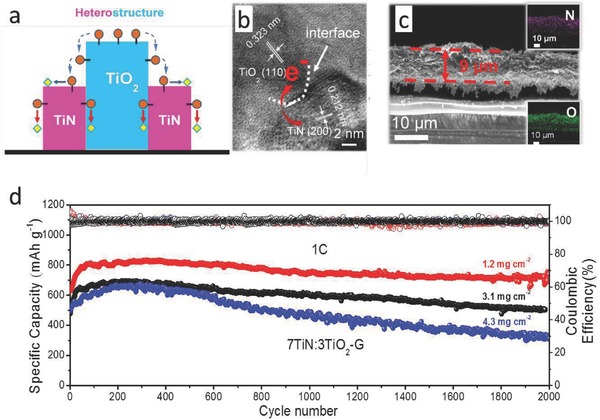
a) Schematic of the conversion process of LiPSs on the TiO_2_–TiN heterostructure. b) High‐resolution TEM images of the TiO_2_–TiN heterostructure. c) SEM image of the cross‐sections of a TiO_2_–TiN/G coating layer, and the insets are elemental distribution maps of N and O. d) Cyclic stability of Li–S batteries with TiO_2_–TiN/G coating layer at 1 C for about 2000 cycles. Reproduced with permission.^43^ Copyright 2017, Royal Society of Chemistry.

Most recently, our group proposed a heterostructure design to construct a highly active interface for realizing a smooth trapping–diffusion–conversion of LiPSs toward ultralong life Li–S batteries. **Figure** **9** presents a typical example for a heterostructure built with the twinborn TiO_2_–TiN that was synthesized by a simple reaction between TiCl_4_ and urea and combines the merits of highly adsorptive TiO_2_ and conductive TiN.^43^ The smooth interface between the twinborn TiO_2_ (for trapping) and TiN (for catalytic conversion) helps achieve an effective trapping, fast diffusion, and catalytic conversion of LiPSs to insoluble Li_2_S_2_/Li_2_S. With loading such a heterostructure onto the graphene substrate as an interlayer structure, the assembled battery delivered high capacity retentions of 73% and 67%, respectively, for the sulfur loading of 3.1 and 4.3 mg cm^−2^ after 2000 cycles under 1 C. One step forward, we successfully constructed an in‐plane graphene–TiC heterostructure by the in situ growth of TiC on a graphene plane that not only acts as the 2D growth template but also a carbon source for TiC formation.^44^ The naturally formed in‐plane interface greatly decreases the diffusion barriers of ion/electron to guarantee a more effective trapping and conversion of LiPSs, expecting a superhigh performance Li–S battery with an optimized preparation. In summary, constructing a smooth out/in‐plane interface to simultaneously realize a fast trapping and catalytic conversion of LiPSs is a promising way to produce an ultralong life Li–S battery.

## 5 Conclusions and Perspectives

For Li–S batteries, the slow conversion between soluble LiPSs and insoluble Li_2_S_2_/Li_2_S is the main cause for the shuttling of LiPSs in the electrolyte, which severely reduces the energy efficiency and cycling performance. Thus, an ideal sulfur host is required to possess not only a strong affinity for LiPSs but also the ability to propel LiPSs‐Li_2_S_2_/Li_2_S conversion, which is expected as the ultimate remedy for suppressing the shuttle effect. This can be realized by the introduction of catalytic materials including catalytic metal‐based materials (metals and their oxides, sulfides, nitrides) and some metal‐free materials with catalytic activities as shown in **Table** **1**. Most of these materials have been widely used and investigated in the desulfurizing industry, and this suggests that the catalysts that promote sulfur‐based compound conversion in traditional chemical reactions may provide a new way to catalytically promote LiPS conversion in the Li–S battery. At the current stage, identifying the catalytic mechanism is expected to provide a design principle for the catalytic sulfur hosts. Thus, in situ characterizations and theoretical calculations are greatly needed to help understand the conversion process between LiPSs and Li_2_S_2_/Li_2_S.

Several key points for high‐efficiency catalyst design for Li–S batteries must be considered.

(1) The catalyst should have a high electrochemical active surface area, and thus, 2D materials with a more accessible surface and 3D materials with network structures are needed to improve the catalytic activity and efficiency. This not only decreases amount of catalyst needed in the electrode to improve the electrode energy density but also provides a large surface area to realize the uniform adsorption of LiPSs and deposition of the Li_2_S_2_/Li_2_S discharge products.
(2) The catalyst should have high conductivity and the structure should be beneficial to ion transport on its surface because the migration and coupling of ions and electrons are necessary to realize the fast conversion of LiPSs. Besides, the poor conductivity of the sulfur and Li_2_S_2_/Li_2_S also calls for highly conductive hosts to ensure good sulfur utilization and decrease the polarization.
(3) For practical applications, low‐cost catalysts such as metal oxides are highly recommended. But due to their low conductivity, hybridization with carbons is required, and thus, the structure of these hybrid hosts needs to be further optimized to realize a high sulfur loading and high catalytic performance in the sulfide–polysulfide transformation.
(4) Designing an out/in‐plane heterostructure, which is built with interfaced highly adsorptive component and catalytic component, is also an effective way to realize a smooth trapping–diffusion–conversion of LiPSs and finally obtain an ultralong life Li–S battery.

In summary, increasing the speed of transformations between the captured LiPSs and the insoluble Li_2_S_2_/Li_2_S is key to suppressing the shuttle effect, and combining the “positive” catalytic effects with the “passive” approaches of physical and chemical confinement shows a much more promising way to solve the shuttling of LiPSs, which is expected as a final solution to the development of high performance and practical Li–S batteries.

**Table 1:** A brief summary of the reported catalytic materials for Li–S batteries

Materials with catalytic effect		Morphology	Sulfur loading [mg cm^−2^]	Capacity retention (100 cycles)		Ref.
				Without catalysts	With catalysts	
Metals	Pt	Nanoparticle	1.21	≈60% (0.1 C)	71.7% (0.1 C)	18
	Co	Nanoparticle	1.3–1.6	N/A	≈68% (0.1 C)	20
Metal oxides	MnO_2_	Nanosheet	0.7–1.0	N/A	≈93% (0.2 C)	21
	Fe_2_O_3_	Nanoparticle	1.0	≈70% (2 C)	≈86% (2 C)	11
	La_2_O_3_	Nanoparticle	N/A	≈60% (0.2 C)	≈68% (0.2 C)	22
	Ti_4_O_7_	Nanoparticle	1.5–1.8	≈75% (0.5 C)	90% (0.5 C)	23
Metal sulfides	CoS_2_	Micrometer‐sized cluster‐like morphology	0.4	≈65% (0.5 C)	≈85% (0.5 C)	28
	VS_2_	Particle	0.9–1.3	≈73% (0.5 C)	≈88% (0.5 C)	12
	WS_2_	Few‐layer flakes	≈1.24	N/A	≈93% (0.5 C)	30
	MoS_2_	Nanoflake	≈1.5	≈50% (0.5 C)	≈73 (0.5 C)	33
	Co_3_S_4_	Nanotube	2.0–4.0	≈45% (0.5 C)	≈85% (0.5 C)	32
Metal nitrides	WN	Nanoplate	N/A	N/A	≈40% (0.1 C)	35
	VN	Nanoribbon	≈3.0	47% (1 C)	≈94 (1 C)	13
	TiN	Particle	1.2	55% (0.2 C, 50 cycles)	70% (0.2 C, 50 cycles)	36
Metal‐free polar materials	Phosphorene	Nanosheet	3.3	≈70% (1 C)	≈100%(1 C)	38
	N‐doped carbons	Particle	2.5	≈50% (0.1 A g^−1^, 70 cycles)	N/A	40
	p‐C_3_N_4_	Particle	0.6	≈45% (0.2 C)	≈67% (0.2 C)	42
Heterostructures	TiO_2_–TiN	Nanoparticle	1.2–4.3	N/A	≈100 (1 C)	43
	Graphene–TiC	Nanosheet	1.1–1.4	≈75% (1 C)	≈100% (1 C)	44

## Conflict of Interest

The authors declare no conflict of interest.
